# Theranostics aspect of extracellular vesicle in cancer liquid biopsy

**DOI:** 10.1016/j.jlb.2024.100139

**Published:** 2024-01-05

**Authors:** Shivani Ravipati, Arghya Nath, Sumitaksha Banerjee, Harendra Kumar, Vindhya Vasini Lella, Swarup Sonar, Dattatreya Mukherjee

**Affiliations:** a Dr. Pinnamaneni Siddhartha Institute of Medical Sciences and Research Foundation, Chinna Avutapalle, Gannavaram, Andhra Pradesh, 521286, India; b ICMR-DHR Viral Research & Diagnostic Laboratory, Burdwan Medical College, Burdwan, India; c Burdwan Medical College and Hospital, Burdwan, India; d Dow University of Health Science, Karachi, Sindh, 74200, Pakistan; e Konaseema Institute of Medical Sciences and Research Foundation, Chaitanya Health City, NH216, Amalapuram, Andhra Pradesh, 533201, India; f Genpact, Badshahpur, Sector 69, Gurugram, Haryana, 12210, India; g Raiganj Government Medical College and Hospital, India

**Keywords:** Extracellular vesicle, Cancer, Exosomes, Cell therapy

## Abstract

Recent advances in cancer treatment emphasize the pivotal role of extracellular vesicles (EVs), especially exosomes (a subpopulation of EVs, originating from endosomes). Exosomes are signaling molecules in the cellular system. Its molecular signature displays the status of the cell (cell healthy or undergoing any clinical complication). Exosomes are frontiers of upcoming cancer theranostics era. This article highlighted cancer exosome complex interlink, the advantage of exosome-base liquid biopsy, the effectiveness of exosome-based cell-free therapy compared to cell therapy, complications of exosome research, and its solution. Hope, this article motivated cancer researchers to explore the exosome-based precision oncology era.

## 1 Introduction

Cancer is the most complicated health crisis worldwide. Current time extracellular vesicle-based (EVs) cancer investigation are the most exciting domain of cancer research. EVs generally play a vital role in cell-to-cell communication. It transports several bioactive molecules such as DNA, RNA, proteins, and lipids [[1], [2], [3]]. It is the source of cancer biomarkers in cancer liquid biopsy and on the other hand, it is a potential therapeutic tool [4,5]. EVs-based cancer liquid biopsy is an effective approach in cancer research. It domain of cancer research also faces some challenges such as EVs heterogeneity, and standard isolation protocol. Therapeutic development prospection EVs open a new horizon in cancer therapeutics. Multiple sources of exosomes are used as a therapeutic in cancer (stem cell, plant exosome, engineered exosome, chimeric exosomes, CAR-T exosomes, and immune cells-derived exosomes) [6]. These exosome-based cancer therapeutics show promising activity in inhibiting tumor growth. EVs-based cancer therapeutics are a cell-free approach because of it they overcome several limitations of traditional therapy (EVs therapeutics are more biocompatible, less toxic, able to cross biological barriers, and specify drug delivery). Exosome-based liquid biopsy is a promising platform for cancer screening. This article explores EVs and cancer interlink, the significance of EVs in liquid biopsy, and EVs and cell therapeutic comparison.

## 2 Biogenesis of exosomes

**Fig. 1:**
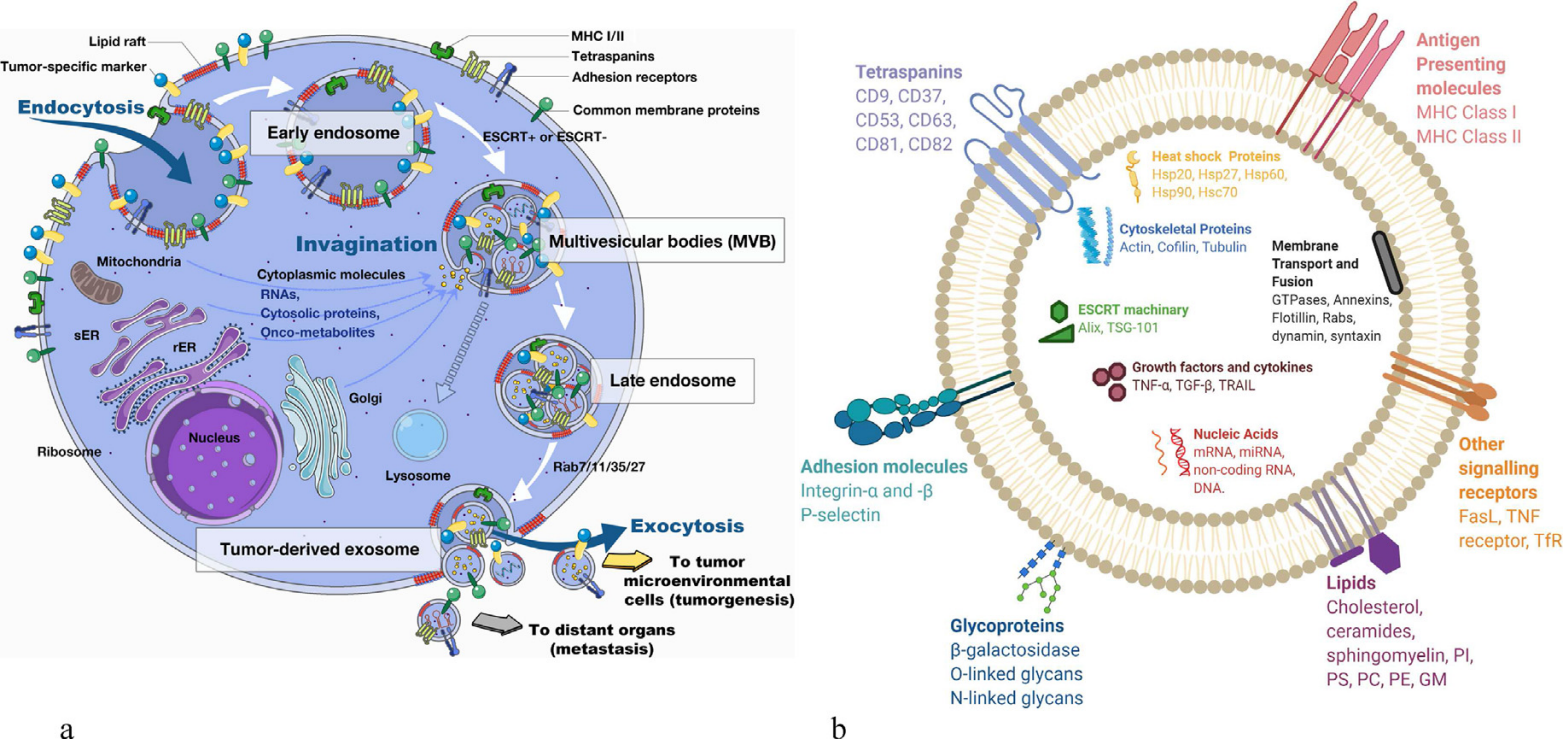
Exosome biogenesis (a) and components (b). (Reproduced with permission under Creative Commons CC BY 4.0 license from ref. [8] Copyright 2018, and for b from ref. [41] Copyright 2021 The Authors).

Biogenesis of exosomes is the multistage maturation process. Inward budding of the plasma membrane forms the early endosome. The endosomal membrane changes and maturation occur simultaneously, which leads to the invagination of the endosomal membrane into the intraluminal space, which forms the late endosome with multiple intraluminal vesicles (ILVs) called multivesicular bodies (MVBs). SNARE proteins, Rab GTPases, and a few other proteins release exosomes into the extracellular space as a result of the fusion of MVBs with the plasma membrane. ILV biogenesis can be ESCRT-dependent or ESCRT-independent (Fig. 1). ESCRT [5] (Endosomal sorting complex required for transport) involves the assembly of four ESCRT complexes that coordinate cargo sorting and membrane deformation to generate ILVs. The AAA ATPase complex Vps4 plays a role in ESCRT disassembly and membrane remodeling. Molecular players like ALIX and syndecans participate in this pathway as well. The ESCRT-independent pathway, on the other hand, involves tetraspanins, S1P1 receptors, and ceramide, which contribute to membrane deformation and the formation of ILVs without the use of ESCRT complexes. These pathways collectively illuminate the intricate mechanisms for the release of exosomes [7]. Similarly, tumors also form exosomes. Tumor-derived exosomes that show tetraspanins and microRNA deliver the tumor cells to normal fibroblasts or endothelial cells, leading to metastasis of cancer [8]. Cell-secreted exosomes are isolated through several methods such as ultracentrifugation, co-precipitation approach, size-exclusion chromatographic method, etc. [9].

## 3 Exosomes and cancer

**Fig. 2:**
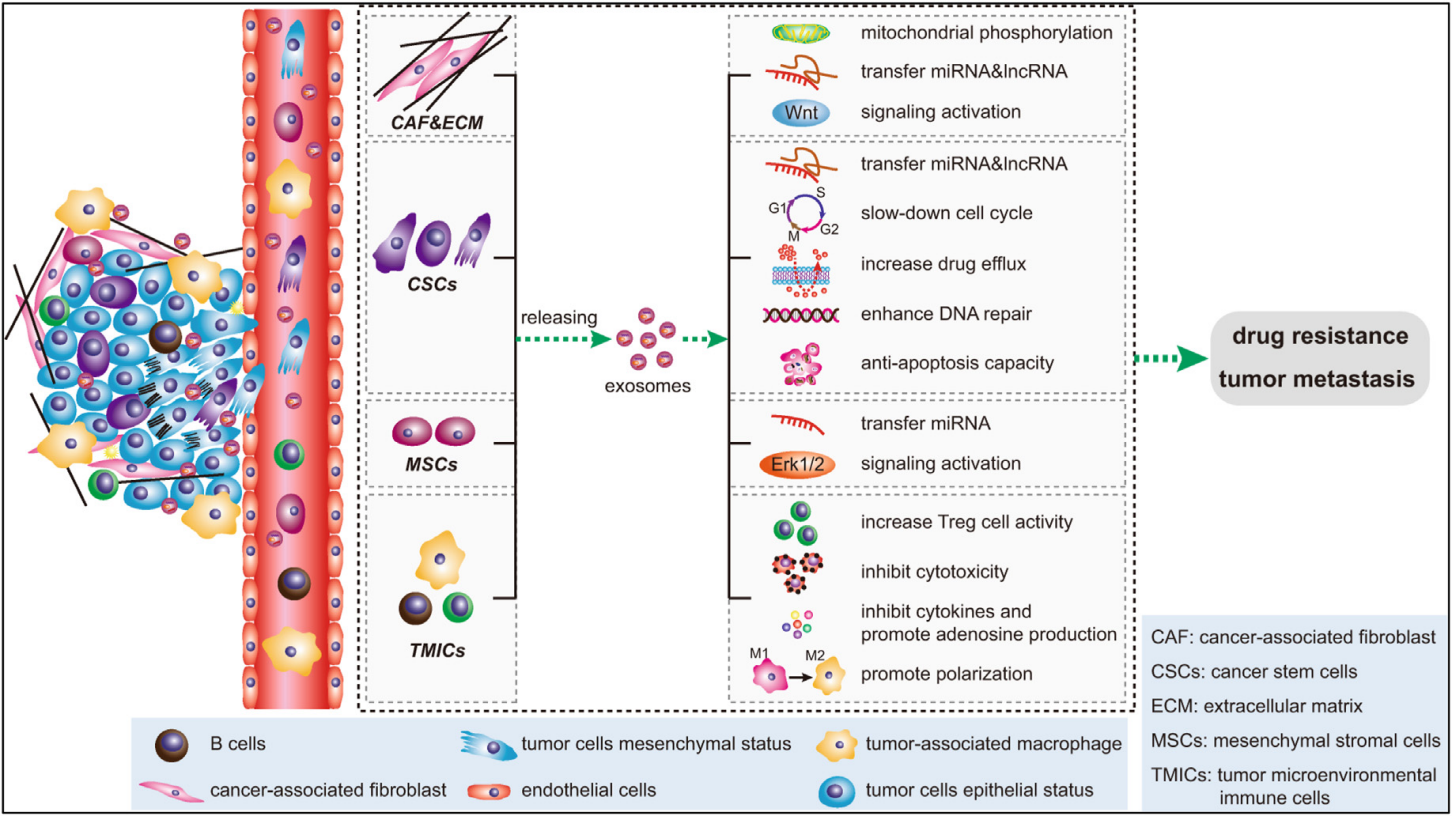
Role of exosome cancer development (Reproduced with permission under Creative Commons CC BY 4.0 license from ref. [42] Copyright @2020 The Authors).

Exosomes are derived from tumors as well (TEXs), which, through various mechanisms. It plays a vital role in cancer progression and development (Fig. 2) [10]. It also has a vital interlink cancer stem cell-based therapeutic resistance development [11]. These TEXs can be biomarkers for cancer detection [10]. There are several sources of exosomes used for biomarkers investigation such as blood, plasma, serum, saliva, etc. [5,12]. On the other hand, multiple exosome sources used for therapeutic development such as mesenchymal cell-derived [13], plant-derived [14], immune cell-derived [15], TEXs-based [16,17], chimeric exosome [18], CAR-T cell-derived exosomes [19], and CRISPR technology combine exosomes [20]. Exosomes are advantageous for being efficient as drug delivery vehicles because of their nano size, low immunogenicity, ability to bear specific surface proteins like integrins, and double-layered lipid membranes for protection and easy transfer between cells [21]. MiRNAs, siRNAs, small molecules, and proteins are the types of drugs that exosomes can deliver [22]. Exosomes can transfer anti-cancer drugs to treat various cancers, such as gastric [22,23], lung [24], brain [25,26], pancreatic [27], leukemia [28], cervical [29], and prostatic cancers [30]. In cancer, ESCRT-independent pathways are associated with exosome production [5]. In extra during cancer TEXs also linked with Cancer Cachexia [31].

## 4 Liquid biopsy vs. Tissue biopsy

**Fig. 3:**
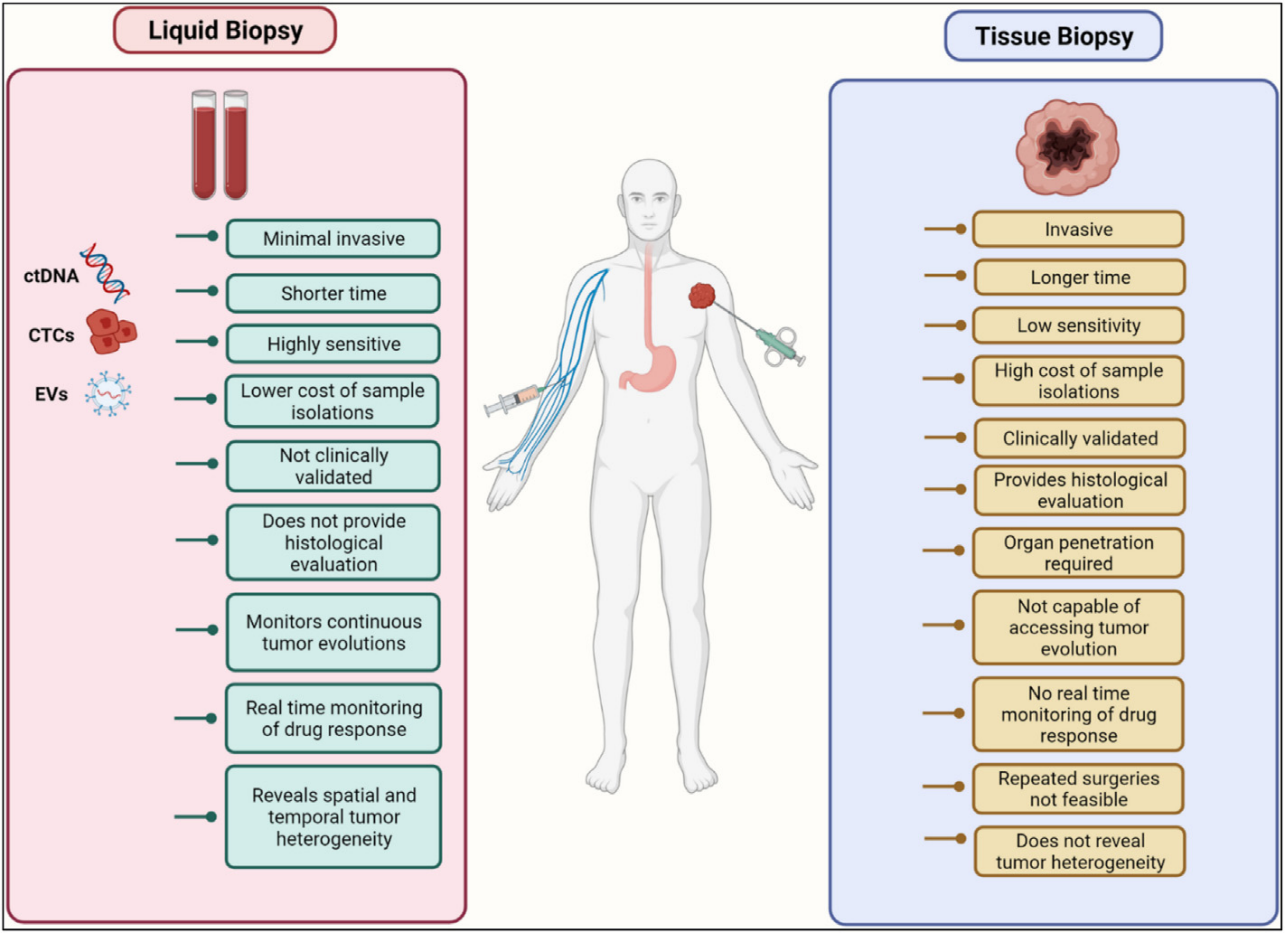
Difference between liquid biopsy Vs. tissue biopsy (Reproduced with permission under Creative Commons CC BY 4.0 license from ref. [32] Copyright @ 2022The Authors).

Cancer screening needs a promising solution for early detection support Several treatment approaches fail due to early detection. Liquid biopsy is more efficient compared to tissue biopsy [32] (Fig. 3). This method also has some limitations, the most significant drawback of liquid biopsy is the requirement for a tissue sample to achieve an initial histologic diagnosis. Assays for ctDNA give little insight into numerous tumor features relevant for grading and staging and only provide an indirect indication of total tumor load [1]. Liquid biopsy assay is that it may miss critical genetic alterations in early disease states simply because ctDNA from a particular tumour may exist in extremely low concentrations. This may result in a delay in the diagnosis and administration of crucial life-saving therapeutics. Furthermore, in some cases, age-related clonal hematopoiesis of indeterminate potential (CHIP) can interfere with ctDNA testing and cause incorrect interpretation of results, leading to inappropriate therapeutic decisions for patient management. Exosome-based Liquid biopsy transforms this method in more effective ways. Exosomes have showed better benefits in liquid biopsy than CTCs and ctDNA. First, the existence of significant volumes of exosomes (109 particles/mL) in biofluids makes collecting vesicles relatively simple, whereas just a few CTCs occur in 1 mL blood samples [3]. Exosomes are released by live cells and contain a wealth of biological information from their parents. As a result, exosomes are more representative than ctDNA, which only represents information from apoptotic or dead tumor cells [3,4]. The reason for their lipid bilayers, exosomes are fundamentally stable and may circulate in healthy settings, even in severe tumor microenvironments. Because of the excellent biological stability, specimens for exosome separation and detection can be stored for an extended period [33]. Current scenario exosome-based liquid biopsy develops this platform next level and the combination of several innovative nanotech-based approaches makes it more effective and accurate [34].

## 5 Exosome vs cell therapy

Exosome therapy demonstrates several advantages compared to cell therapy, making it a promising option for cancer therapy. Exosome therapy vs. Cell therapy comparison summarized in Table 1.

**Table 1:** Exosome Therapy vs Cell Therapy.

Terms	Exosome therapy	Cell therapy	References
Safety concerns	No ability to self-replicate, thus eliminating concerns about potential tumor formation post-transplantation. Less immunogenic	Self-replicates, so it can be prone to cancer development. More immunogenic than exosomes	[35,36]
Stability and storage	Stable for long-term storage, even under lyophilization, freezing, or at room temperature, without compromise on efficacy	Requiring specific, harsh storage and transportation conditions	[37]
Engineering possibilities	Can be engineered or modified to display specific molecules or carry drugs as cargo for targeted drug delivery or vaccine development.	No engineering possibilities	
Regulatory considerations	Free of ethical concerns	May require ethical concerns as sometimes its production involves the destruction of embryos.	
Administration flexibility	Can be administered through various routes, such as inhalation for brain tumors, enabling treatment for specific organs	Rigid routes	[38]

Additionally, recent research progress indicates that exosome-based therapeutic development faces several limitations isolation protocol, heterogeneity [39,40], large-scale production, and less toxicological investigation. Exosome-based clinical research develops a smart platform called single exosome profiling and exosome barcoding approach [34] which can efficiently exosome profiling from clinical samples.

## 6 Conclusion

Extracellular vesicles (EVs) therapy is a cell fee approach. This exciting approach needs some time to explore several facts about EVs therapeutic such as toxicity, stem cell-derived exosome dual nature in cancer, TEXs therapeutic application under a question mark, and plant exosome still safe with low toxicity. In the future, engineered or modified EVs will become a game changer in cancer therapeutic competition. Other hand EVs-based liquid biopsy become an efficient method for early cancer detection. Hope, EVs-based cancer research develops an effective and affordable solution for cancer treatment.

## Availability of data and materials

Data sharing does not apply to this article as no datasets were generated or analyzed during the current study.

## Funding

There is no funding for this study.

## Declaration of competing interest

The authors are declaring no conflict of interests.
